# The Book World of Medicine and Science

**Published:** 1897-04-10

**Authors:** 


### The Book World of Medicine and Science.

Forensic Medicine and Toxicology. A Manual for Students. By C. O. Hawthorne, M.B., Fellow (by examination) of the Faculty of Physicians and Surgeons, Lecturer on Materia Medica and Therapeutics, Queen Margaret College, University of Glasgow; Assistant Physician to the Western Infirmary, Glasgow. Second edition. (Glasgow: A. Stenhouse, College Gate, Hillhead, 1897. Price 4s. 6d., 174 pages, 5 illustrations.)

This book has gained in its second edition by being slightly revised and a few illustrations introduced. It has already taken its place as a compact and reliable book for those studying for examinations, and it may be safely prophesied that a third edition is not far off.

